# A Guide to Diet-Microbiome Study Design

**DOI:** 10.3389/fnut.2020.00079

**Published:** 2020-06-12

**Authors:** Abigail J. Johnson, Jack Jingyuan Zheng, Jea Woo Kang, Anna Saboe, Dan Knights, Angela M. Zivkovic

**Affiliations:** ^1^BioTechnology Institute, College of Biological Sciences, University of Minnesota, Minneapolis, MN, United States; ^2^Department of Nutrition, University of California, Davis, Davis, CA, United States; ^3^Department of Computer Science and Engineering, University of Minnesota, Minneapolis, MN, United States

**Keywords:** microbiome, diet, dietary intake, study design, methodology, personalized nutrition

## Abstract

Intense recent interest in understanding how the human gut microbiome influences health has kindled a concomitant interest in linking dietary choices to microbiome variation. Diet is known to be a driver of microbiome variation, and yet the precise mechanisms by which certain dietary components modulate the microbiome, and by which the microbiome produces byproducts and secondary metabolites from dietary components, are not well-understood. Interestingly, despite the influence of diet on the gut microbiome, the majority of microbiome studies published to date contain little or no analysis of dietary intake. Although an increasing number of microbiome studies are now collecting some form of dietary data or even performing diet interventions, there are no clear standards in the microbiome field for how to collect diet data or how to design a diet-microbiome study. In this article, we review the current practices in diet-microbiome analysis and study design and make several recommendations for best practices to provoke broader discussion in the field. We recommend that microbiome studies include multiple consecutive microbiome samples per study timepoint or phase and multiple days of dietary history prior to each microbiome sample whenever feasible. We find evidence that direct effects of diet on the microbiome are likely to be observable within days, while the length of an intervention required for observing microbiome-mediated effects on the host phenotype or host biomarkers, depending on the outcome, may be much longer, on the order of weeks or months. Finally, recent studies demonstrating that diet-microbiome interactions are personalized suggest that diet-microbiome studies should either include longitudinal sampling within individuals to identify personalized responses, or should include an adequate number of participants spanning a range of microbiome types to identify generalized responses.

## Introduction

**Figure 1:**
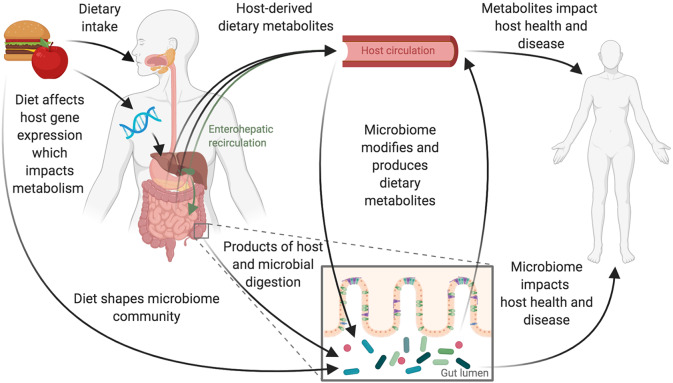
Diet and the gut microbiome interact to influence host health. Dietary intake and the gastrointestinal microbiome interact to affect host physiology and disease. These interactions are complex and likely depend on interplay between the host's genetics and immune system and the composition and function of the gut microbiome. Consumption of foods begins the digestive process, leading to the production of host-derived metabolites primarily absorbed through the stomach and small intestine. Host genetics impacts this metabolism and can also be influenced by diet with some diet-gene interactions altering host gene expression. Food and dietary products that reach the large intestine can shape the microbiome directly. These products are also acted upon by microbes to produce microbially derived metabolites. Host-derived metabolites that have previously entered host-circulation may also pass into the large intestine via biliary enterohepatic recirculation or by transport across the gut epithelium resulting in additional opportunities for the host and microbes to jointly act upon dietary derived metabolites.

Microbiome features and metabolites have been increasingly linked to states of health and disease (1, 2), and diet is appreciated as one of the key drivers of this relationship. The microbes residing in the human gastrointestinal tract depend on their hosts for sources of fermentable substrate. Microbes metabolize the end-products of human digestion and indigestible dietary substrates to produce a wide variety of diet-derived and secondary metabolites. The microbes themselves and their metabolites signal the immune and nervous systems through known and unknown mechanisms, ultimately affecting human physiology, and disease development or progression. Unlike the known relationships between the essential nutrients and disease, which focus on single-nutrient relationships such as that between vitamin C and scurvy, these new diet-microbe-disease, and diet-metabolite-disease relationships are more complex. Microbe-disease relationships likely depend on the production of diet-derived metabolites such as short chain fatty acids (SCFAs) or on the production of secondary metabolites created by microbes prior to entering host circulation (Figure 1). While mechanisms have been identified for certain unique diet-microbe-metabolite relationships (3, 4) and a growing body of evidence suggests that the microbiome is playing an important role in drug metabolism (5), the complexity of the changing landscape of each individual's gastrointestinal tract has proven difficult to study. With each individual person's microbiome acting as an ecosystem, the majority of mechanisms explaining exactly how diet alters the microbiome and conversely how the microbiome alters dietary inputs to impact health remain uncharacterized.

High interindividual variation in both dietary intake and microbiome composition contribute to our poor understanding of the relationships between the foods we eat and how they impact the bacterial communities that reside within our bodies (6). It is difficult to control for diet and microbiome covariation in observational and interventional studies. In this article we discuss the limitations and complexity involved in planning dietary intervention studies with microbiome outcomes. We identify variables that should be considered, controlled for, and recorded in study designs. Finally, we attempt to make practical suggestions for study designs moving forward that appropriately incorporate and control for nutritional factors such as food intake and dietary patterns.

## A Brief Review of Diet-Microbiome Interactions

Past efforts to understand diet-microbiome relationships have linked dietary features to microbial composition (7, 8), but few diet-microbe-metabolite pathways have been decoded. Most of the exploration of diet-microbe-metabolites has been focused on SCFA production from fiber. However, other metabolites are gaining increased interest. Products of bacterial proteolytic fermentation from dietary proteins have been linked to negative health conditions such as diabetic kidney disease (9) and insulin resistance in individuals without diabetes (10). A large focus on the research in this space has centered on the microbial production of trimethylamine (TMA) from host dietary choline, which after absorption and conversion to trimethylamine N-oxide (TMAO), becomes a circulating microbe-host co-metabolite that is associated with increased risk for cardiovascular disease (3). Blocking microbial TMA production in a mouse model has been demonstrated to reduce atherosclerotic lesion development (11). The production of secondary bile acids by microbes have also been linked to health outcomes. For example, the relationship between cream-derived saturated fat, host production of taurocholic acid, and *Bilophila wadsworthia* has been identified in animal models as potentially important for the development of inflammatory bowel diseases in genetically susceptible individuals (12). Microbial production of secondary bile acids may also impact host metabolism by producing ligands that bind to the farnesoid X receptor (FXR) (13) and the G protein-coupled bile acid receptor-1 (also known as the Takeda G-protein-coupled receptor-5[TGR5]) (14). A large amount of work remains to be done in this space for these relationships to be exploited for their therapeutic potential because the majority of metabolites measured by untargeted mass spectroscopy remain uncharacterized (15).

A growing body of literature suggests that microbial responses to foods are personalized. For example, efforts to improve blood glucose control based on microbiome features suggest that food-microbe pathways could improve health in a personalized manner (16). The abundance of *Prevotella*, in particular, has been described as a way to differentiate between responders and non-responders with improved glucose metabolism following consumption of bread high in barley fiber (17, 18). Similar findings of responder and non-responder microbiome features have been reported after consumption of whole grain sourdough or white bread (19). Microbial responses to fiber-specific dietary interventions have also revealed responder and non-responder phenotypes related to the production of SCFAs (20, 21). Beyond treating dysbiotic states and predicting biochemical responses to food, changing diet or providing prebiotics in a personalized way could also theoretically change the microbial community to be more accepting of new members from a fecal transplant or after administration of probiotics. The future of personalized nutrition will likely need to harness all of these mechanisms, but the knowledge base needed to design microbiome-informed, personalized nutrition interventions is currently limited.

Diet is attractive as a modifier of gut microbiome composition because it itself is modifiable. If we can modify diet to modify the microbiome or the metabolites produced by the microbiome, then we can potentially prevent or modulate disease outcomes. There are two ways that dietary intake can interact with the microbiome to impact health. First, specific diets, dietary patterns, food components, and foods may change gut microbiome composition in a predictable way. For example, many studies have found an enrichment in protein and fat metabolizing microbial species or genes in Western diets, which are enriched in animal products and deficient in fiber, and conversely an enrichment in saccharolytic microbes in diets high in fermentable fiber (22–24). Second, diet-derived metabolites may themselves be modified by sets of microbes or individual microbial strains and affect host physiology (25). For example, the soy isoflavone daidzein is a phytoestrogen that can be metabolized to equol by gut microbes, and this gut microbe-derived metabolite has significantly stronger estrogenic activity than its precursor (26). Although equol production is common in animals, only ~30% of humans harbor microbes that convert daidzein to equol, with important implications on the estrogenic potential of soy-derived isoflavones on human health (26). However, while diet may predictably change some aspects of the microbiome and while certain dietary components may be predictably modified by certain microbes harbored by different groups of individuals, many food-microbe interactions are variable and dependent on the individual (6, 16).

Altering the microbiome through dietary intake is not a new concept. One hundred years ago John C. Torrey stated that, “It is now well-known that diet exercises a profound influence on the determination of the types of bacteria developing in the intestinal tract (27).” Scientists of the early 1900s accepted that the intestinal flora, as they referred to what we now call the microbiome, changed quickly in response to changes in diet. These findings were built on a foundation of animal studies (28), but they incorporated minimal evidence from humans (29). Likewise, modern diet-microbiome understanding has grown out of strong foundations in animal models—primarily mice (12, 30)—with other non-human primates also contributing to our understanding (31). Increasingly, there is a need to translate findings from animal models to free-living humans and recent work has made strides toward this effort. The body of diet-microbiome literature has recently been reviewed elsewhere (15, 25, 32–34).

## Current Diet Assessment Practices and Their Limitations in Diet-Microbiome Studies

The advent and increasing availability and affordability of sequencing technology has resulted in an explosion of diet-microbiome literature. This is easily illustrated with a PubMed search of “diet” and “microbiome.” In 2009 there were 100 papers published and in 2019 there were 2,204 papers published that were identified using these search terms. As of January 2020, there are 9,544 papers that are returned on PubMed using these terms, and of those over half were published in the last 3 years. This increase in publications has been accompanied by a growing awareness of the limitations we face while attempting to measure and analyze the highly complex interactions between microbes, dietary exposures, and host phenotypes.

Measurement of dietary intake remains particularly challenging. While the methods for collection and analysis of microbiome data have improved over the past decade, there has been little change in the analysis and collection of dietary data with many human studies relying on food frequency questionnaires (FFQs) or self-administered single day food records or 24-h dietary recalls. Each of these methods is prone to reporting errors and is associated with advantages and disadvantages, as has been reviewed extensively elsewhere (35). Importantly, while certain dietary assessment approaches may be adequate for estimating total caloric intake, dietary diversity, or the intake of certain foods and food categories, they may not capture the level of detail needed to discover relationships between diet and the gut microbiome. Although improved methods for the collection and assessment of diet in microbiome studies are desperately needed to address these issues, the development and adoption of new dietary assessment techniques will take time. In the meantime, careful consideration of key variables when planning studies and integrating dietary data with microbiome outcomes is recommended.

Accurately measuring and assessing dietary intake using self-report and nutritional biomarkers is challenging (36, 37). When choosing a dietary assessment technique for a microbiome study the method selected will ultimately impact the research questions that can be answered using the data. Like most research decisions, to assess diet it is necessary to weigh competing options in terms of time, quality, and cost. In this case, time includes both investigator time for collection and review, and participant response time and burden due to effort expended to record diet. Participant time is influenced by the timeframe of dietary record keeping. Low-burden dietary assessments include the administration of a single 24-h recall or record or a single FFQ to capture dietary history over the preceding weeks, months, or years. More time-intensive longitudinal dietary assessments, using detailed daily records over an extended period of weeks, months, or even years have a high participant burden. Quality relates to how closely the data from the dietary assessment accurately captures and reflects actual dietary intake, and how well the data capture the level of detail necessary for microbiome-related outcomes such as the inclusion of microbially-important diet-derived chemicals in nutrient databases (38). Cost is also multifactorial, including the cost of any nutritional software or physical measurement collection devices like scales and measuring cups, to the cost of trained personnel to collect or enter daily 24-h recalls, and the cost needed to pay participants to encourage participation and complete record keeping. Typically, researchers can only pick two of these three competing interests—time, quality, or cost. Therefore, researchers usually choose to minimize participant time burden and overall cost at the expense of quality. What this means in practice is that research teams frequently use FFQs or dietary screeners instead of more time- and cost-intensive methods like multi-day diet records or recalls administered by trained personnel.

Dietary measurement by FFQ has both advantages and disadvantages (39, 40). The primary advantage is that FFQs are convenient. They are very easy to administer and take less participant time than other methods. A study participant simply indicates the frequency at which they consume specific foods and how much they consume, and these answers are used to estimate total caloric intake, as well as the intake of the major macronutrients and micronutrients. However, because FFQs were developed to quantify broad dietary patterns or indices of healthy eating, they are limited in a number of ways and cannot capture diet as accurately as other methods (41). While far from optimal, dietary patterns estimated by FFQ have provided some insight into the way habitual diet contributes to microbiome composition; long-term FFQ-determined nutrient intake has been associated with microbial composition, and relationships found between FFQ-determined dietary patterns and the abundance of microbial genera (7, 42–44). These findings have been supported by research showing that changing dietary patterns, either experimentally or through natural experiments that take advantage of seasonal eating habits or immigration, affect microbiome composition (45–47). However, most FFQs are not designed to provide data that can link specific foods to specific changes in microbial species composition or functional pathways.

Despite the identification of some signals from microbiome studies that use FFQs for dietary analysis, the technique is simply not specific enough to untangle the complex relationships between foods and the microbiome. We have shown previously that microbiome composition more closely covaries with food intake, not nutrient intake (6), indicating that reliance on existing nutrient composition variables is insufficient and that foods themselves are important when exploring diet-microbiome covariation. However, even well-conducted 24-h recalls and food records fail to sufficiently capture the complexity of foods in ways that are meaningful to microbes. For example, one brand of bread may include 7 different whole grains as well as nuts and seeds, while another brand may be made with sprouted wheat. During diet entry both of these breads may be coded as whole-wheat bread and therefore downstream analysis will treat them as identical when they are different. This variation could contribute to some of the personalized diet-microbiome results that have been reported with respect to blood glucose response (16, 19).

In instances where researchers recognize the need to collect more finely resolved dietary information, it is common in the microbiome literature to read about techniques that use researcher-developed food questionnaires, independently created app-based collection methods (16, 48), and consumer-facing tools for nutrient analysis rather than using validated techniques for dietary collection or nutrient analysis tools developed with a research focus (23). When collecting 24-h recalls or asking participants for 24-h food records, inclusion of properly trained staff can improve data quality. At a minimum, participants recording their intake should be trained using detailed examples showing the level of detail necessary to complete an accurate record. This should include a discussion of serving sizes, ingredient specificity, preparation methods, and the inclusion of commonly forgotten foods and additives. All records should be reviewed with the participant with a focus on identifying misreported or commonly forgotten foods. Methods for dietary intake assessment are improving and technology using computerized recalls and records can greatly reduce researcher burden when collecting dietary data. Regardless of collection method, when records or recalls are coded for analysis using dietary research software, consideration for consistency with data entry, coding, and cleaning of dietary data is important to allow for robust analysis of nutrient composition and food intake downstream.

In the current dietary record collection environment, different dietary collection tools and FFQs rely on different underlying databases, which makes comparison across studies and cohorts difficult, if not almost impossible. Even within the English speaking regions of the Americas, United Kingdom, and Australasia, food records are ultimately mapped back to different databases depending on the software tools used for analysis (49). Each database provides nutrient composition data, but the source of that data varies. The analysis methods for specific nutrients can also vary by database which leads to different nutrient level outcomes in databases from different countries. Beyond nutrient composition, the naming conventions used to identify foods are not identical across databases leading to issues when comparing data collected using different tools. Additionally, food-grouping structures vary, with no universally accepted method adopted across studies, or databases. Efforts to establish a shared food ontology that can harmonize dietary data collected in different global regions and by different tools are in progress, incorporating other food features such as preparation method (50). Food preparation and cooking methods alter the chemical properties of foods, such as the changes that occur to sweet potatoes during cooking, that then impact the effects of that food on the microbiome (51), adding another layer of complexity to measurement of diet. The food matrix (52) is likely to play an important role in the relationship between diet and the microbiome and should also be considered. Specific information such as the ripeness of fruits is not currently captured in any food databases. However, this level of detail may be highly relevant for certain food-microbe interactions. For example, it is known that when bananas are unripe or green the starch contained within them is resistant starch (53), which is fermentable by microbes, whereas in ripe bananas that resistant starch has broken down into simpler starch and glucose molecules, which are absorbable by the host, and no longer provide any fermentable substrate for the gut microbes. Beyond food preparation and ripeness, recent research addressing eating behaviors around ultra-processed foods shows that controlling for energy and macronutrient content alone may be insufficient for dietary interventions (54). Food sourcing, processing or cooking methods (51), additives or emulsifiers (55, 56), artificial sweeteners (57), and conventional or organic farming methods (58) likely also need to be taken into consideration.

In addition to the microbiome changes induced by the biochemical components of foods, foods themselves contain bacteria that affect the gut microbiome. From a health perspective, fermented dairy such as yogurt and cheeses, are the most commonly recognized foods that contain “beneficial” microbes (59). These foods are sources of microbes that can transiently populate the human gut (23). Fresh, non-fermented foods have long been recognized as a source of food-borne pathogens and are the target of public health interventions to prevent the spread of food-borne disease. Despite this recognition, we know surprisingly little about the microbial composition in other non-fermented foods. Recently, crops that are not usually considered to transfer bacteria, such as apples, have been shown to harbor a microbiome that depends on growth and farming practices (58). Indeed, dietary patterns that include more food types, particularly fermented foods like yogurt, contain a higher abundance of microbes relative to less diverse diets (1 × 10^9^ colony forming units [CFU] vs. 1 × 10^6^ CFU in a day's worth of meals) (60). Consideration for the microbial load of a dietary pattern is important because the engraftment of non-pathogenic food-borne bacteria depends, in part, on other dietary components. For example, higher abundances of parmesan-cheese-associated bacteria are present after consumption of milk products (61). Ideally, we need to consider these microbial features of diet when planning and analyzing microbiome-diet studies.

## Considering Day-to-Day Variation in Both Microbiome and Diet

As the number of longitudinal microbiome studies steadily increases in the literature, controlling for within-individual variation is an increasingly important consideration during study design. Here we present reasoning that supports including multiple consecutive microbiome samples per study timepoint or phase, and multiple days of dietary history prior to each microbiome sample whenever feasible; thus capturing longitudinal change within an individual by measuring changes over time or before and after a dietary intervention, while also using repeated sampling around set timepoints to control for within-person variation. To support these recommendations, we provide suggestions for microbiome sample collection, transport, and storage for diet-microbiome studies and explore questions that researchers designing studies in this space should consider.

### Considerations for Sample Size and Consecutive Microbiome Samples

Diet explains around 5–20% of the variation in microbiome (6, 46, 62, 63). In every cohort there is a large amount of variation between individuals that is not driven by diet and likely depends on environmental differences, early life exposures, or immune and other host differences (64, 65). Without establishing a baseline microbiome for each study participant, it is difficult to assess the effects of dietary interventions on the microbiome. Moving toward designs that can account for inter-individual variation is necessary to improve microbiome studies (66). Studies can minimize the impact of inter-individual variation by increasing the number of participants enrolled in a study. The number of participants needed to assess the effects of a particular dietary intervention on the gut microbiome likely varies depending on the research question and anticipated taxon difference in a case-control setting. Study populations of 400–500 individuals are necessary to power case-control microbiome studies to detect differences in dominant taxon proportions of between 5 and 9% (62), suggesting that these sizes should be sufficient to detect differences of dietary origin. This also suggests that a substantial amount of cross-sectional research in the diet-microbiome space may be underpowered to detect dietary impacts on microbiome composition and raises questions regarding when cross-sectional study designs should be used, if at all, to assess diet-microbiome interactions. We expect that in the majority of cases longitudinal studies in which subjects serve as their own control will be more fruitful than cross-sectional studies, and we expect that cross-sectional studies will require large sample sizes.

Increasing sample size will boost power to identify diet-induced microbiome differences but does little to account for the dynamic nature of the microbiome. Increasingly, data support the need for repeated sampling of the microbiome to account for intra-individual variation with 3–5 daily sequential fecal samples in aggregate providing better results over single samples in inflammatory bowel disease cohorts (67) and in healthy individuals (6, 68). Averaging repeated samples and looking at changes within an individual over time allows for greater power to detect microbiome differences by removing within-person noise; thereby reducing the need to dramatically increase sample sizes. In both healthy and disease cohorts, little improvement is seen with the collection of more than 7–9 serial samples (67, 68). The caveat for the collection of 3–5 serial samples per person is that the strongest data for this recommendation comes from cohorts of healthy individuals. While some data from inflammatory bowel disease cohorts suggests this is sufficient to capture variability (67), there are insufficient data for most other microbiome-related conditions where the microbiome may have large compositional swings that are not captured with this density of sampling.

**Figure 2:**
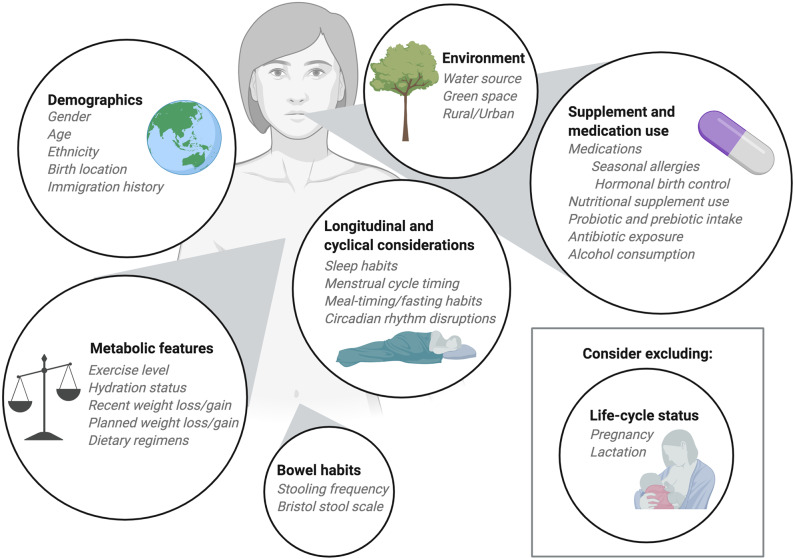
Considerations for participant enrollment and data collection. When planning diet-microbiome studies researchers should take into consideration numerous participant features when determining inclusion and exclusion criteria. Demographic information should be collected with a focus on features and exposures known to impact the microbiome. Information about medication use, including commonly consumed over-the-counter medications should be recorded. Depending on the research question, researchers may choose to exclude participants who consume supplements, prebiotics, or probiotics. Alternatively, researchers may ask participants to maintain consistent use of nutritional products throughout the study period. Recent infection and antibiotic exposures should be considered as part of the inclusion and exclusion criteria as should alcohol consumption. In longitudinal studies, normal cycles including sleep cycles, menstrual cycles, and meal timing or fed/fasted cycles should be collected as covariates. The metabolic status of participants can be an important covariate and, when possible, exercise level and hydration status should be recorded and held constant. For research with metabolic outcomes, exclusion of participants who have recently experienced weight loss or weight gain may be important. Typically, we exclude pregnant and lactating participants from our dietary intervention studies, but in some instances it may be appropriate to include these individuals. Finally, bowel habits should be considered at enrollment and during study participation. When collecting multiple consecutive stool samples from participants the frequency of stooling can impact study retention and complicate study timelines so it can be helpful to interview participants about their usual bowel habits before enrollment.

In addition to considerations regarding sample size and sampling frequency, researchers should take into consideration participant selection as a means to improve diet-microbiome research (Figure 2). When enrolling participants for dietary intervention trials it can be useful to consider the baseline microbiome of participants. In situations where a 1-2 week turn-around from collection to sequence is possible, there are opportunities to consider participant stratification or exclusion by baseline microbiome composition during recruitment. Current sequencing timeframes likely prevent the incorporation of this type of baseline assessment. However, with advances in fast, affordable sequencing this may be possible in the future. If we imagine a future where baseline microbiome-typing is feasible, then it should be possible to select or stratify participants with the goal of including a range of microbiome compositions at baseline to capture the breadth of possible responses to dietary interventions, or the converse goal of selecting only participants with a certain type of microbiome, for example, *Prevotella*-dominant, to increase power. In study designs for dietary interventions where the length of the intervention required makes a cross-over design difficult, stratified randomization or an interspersed treatment design that incorporates baseline microbiome could make parallel arm studies more feasible. As an alternative, selection for a specific baseline microbiome phenotype should improve the ability to assess how interventions depend on the presence of particular microbes. As a thought experiment, we consider the situation of studying a specific prebiotic. A researcher in this situation may want to choose only people who have the targeted microbe that is expected to be enriched by that prebiotic, or to choose people who have a low abundance of fiber-degrading microbes to see how the intervention affects that subgroup. Importantly, microbial phenotyping at baseline would allow for randomization into study arms using a stratification design that incorporates baseline microbiome composition, thereby avoiding the situation where after randomization clusters of individuals with similar microbial compositions are assigned to the same treatment arm (6).

### Strategies for Fecal Sample Collection and Sequencing

Repeated and longitudinal microbiome sampling strategies require careful consideration of fecal sample collection, transportation, and storage to prevent unintentional bias due to the potential for the composition of these living communities to change after collection. Comparisons of fecal microbiome collection, transport, and storage methods have been detailed in several recent publications (69–72). When considering longitudinal studies and repeated microbiome sampling the collection process selected for the study has a large impact on participant compliance and will also dictate the parameters that should be considered in terms of temperature and transport.

Several commercial collection methods are readily available for researchers and others can be easily assembled from standard laboratory supplies. Prior to choosing a collection kit researchers should consider the volume of sample necessary for sequencing and other planned analyses, as well as the conditions and available resources associated with the study design. Swab fecal collection requires very little fecal material and allows participants to easily obtain specimens from used toilet paper or collected stool samples. Swab collection has been used successfully in large scale citizen science projects like the American Gut Project (73). The American Gut Project sampling methodology which uses a swab to take a small amount of fecal material from a stool or soiled toilet paper did not significantly alter microbial diversity and composition as measured by 16S amplicon sequencing compared to other methods (74). However, swabs might not be ideal for studies where larger amounts of fecal material are necessary, for example where metabolomics is required in addition to sequencing (75).

For studies that require a large volume of fecal material, collection of an entire bowel movement can be completed using a stool collection hat or container. The collection of the entire bowel movement may be important for some studies considering that the distribution of microbial species in a single bowel movement is not homogenous (76, 77). Fecal sampling location within a single stool sample may affect the abundance of specific microbes, reflecting the diverse microbial communities residing in small niches throughout the gastrointestinal tract (78). Collection of the entire bowel movement presents significant logistical challenges including the need for study participants to collect and transport large sample volumes. When collecting whole stool samples researchers should work closely with participants to agree upon collection times. Fresh samples should be transported to the research facility quickly where study personnel can process, aliquot, and store the sample. Consideration should be given to the timing of bowel movements and participant or researcher schedules when incorporating whole stool collections into protocols.

For most studies, participant self-collection of a few grams of fecal material into a collection container using a sterile spatula or scoop provides ample material for deep shotgun sequencing or even metabolomics without undue participant or researcher burden. Subsampling of a stool sample can over- or under-represent the relative abundance of certain taxa and under-report low abundance taxa (79). However, the differences in composition relative to whole stool samples are minor compared to inter-individual variability (80); making this a viable collection methodology for clinical studies. Participants can be trained to collect a fecal sample using a stool collection hat and then to transfer a portion of the whole stool sample into one or more tubes. One benefit of this collection strategy is that the participant can transfer fresh samples into one or more small cryovials or microcentrifuge tubes which can be immediately stored in regular freezer boxes; minimizing sample handling and diminishing sample degradation from freeze-thaw cycles (81).

Regardless of which sampling and collection technique is selected, the collection container can be prefilled with preservative, which allows for the sample to be shipped and stored at ambient temperature. If samples are collected without preservatives then they need to be temperature controlled from the point of collection (e.g., placed in coolers with ice packs or dry ice, temporary storage in home freezers) until they can be stored appropriately at −80°C in a research facility. Immediate sample storage at −80°C is considered the gold standard regardless of the presence of a preservative. In most human studies, practical considerations around the timing of bowel movements, location relative to the research facility, or availability of refrigeration requires the use of a preservative to prevent microbial growth in these samples. Preservatives can allow samples to be stored at ambient temperatures for as long as 60 days without impacting technical reproducibility (82); although the exact number of days varies by preservative (74, 83). Commonly used preservatives like 95% ethanol and RNA*later* result in high reproducibility and stability, as do some commercially available kits like the OMNIgene gut kit (71). Many preservatives can interfere with fecal metabolites (84), however when multiple analyses (DNA, RNA, and metabolites) are required from the same sample 95% ethanol may have advantages over other methods (82). Overall, as long as the preservation and collection method is standardized within the study, many methods provide acceptable stability and reproducibility.

Choosing a DNA sequencing technique for the analysis of collected fecal samples is another important consideration. When selecting a method, the desired taxonomic and functional resolution required to address study hypotheses should be carefully considered. The primary sequencing methods available for microbiome classification are 16S amplicon sequencing and metagenomic sequencing. The 16S ribosomal RNA gene is conserved across bacteria and contains nine hypervariable regions (V1-V9) that have amassed changes throughout evolutionary history. Amplification and sequencing of the variable regions within this gene (i.e., V3-V4) (85) allows for a low-cost and rapid way to detect the taxonomic composition of a sample (86). However, this method is relatively low-resolution and does not allow for accurate elucidation of bacterial taxonomy beyond the genus level. Since this method involves the use of primers to amplify the target bacterial gene, bias may also be introduced that affects the outcome of sequencing (87, 88). Therefore, this method would not be desirable when more detailed assessment of taxonomy is desired.

An alternative to 16S sequencing is deep whole metagenomic shotgun sequencing, which involves the sequencing of all microbial genomes in a sample. This method provides resolution to species level of taxonomy, and can also detect eukaryotic species and viruses to provide information on other microbes in a sample (86). Amplification bias is less likely to occur with this primer-free approach, but one downfall is that it is significantly more expensive, which may affect the feasibility of use for larger-scale studies. Shallow shotgun sequencing is an alternative to deep shotgun metagenomic sequencing. This method is significantly cheaper and has been shown to provide accurate resolution of species despite having a lower sequencing depth in comparison to deep sequencing (89). Shallow shotgun sequencing has proven effective in assessing longitudinal covariation of the microbiome with dietary intake demonstrating that this is a viable approach for cost-effective longitudinal diet-microbiome studies (6). Additional approaches for and analysis of microbiome data with a clinical focus have recently been thoroughly reviewed including the considerations for when to include metatranscriptomics and metabolomics approaches in analysis pipelines (90).

### Collecting Multiple Days of Dietary History Prior to Each Microbiome Sample

For observational studies where dietary intake is considered as a confounder for the microbiome outcome of interest, the decision to collect dietary data is driven by different factors than when dietary intake is the exposure of interest. We do not think that all microbiome studies need to collect dietary data. In many well-designed microbiome studies it may not be necessary to collect dietary data and the decision to collect dietary data should be weighed carefully with the researchers' hypotheses and planned analyses. In observational studies where diet will be a clear confounder that cannot be controlled for through other study design parameters, investigators should collect detailed information about diet.

There are additional factors causing personalization of diet-microbiome interactions. Of particular importance is the effect of transit time on microbiome-diet study planning and analysis. Transit time has been shown to affect the microbiome directly. Fukuyama et al. conducted a longitudinal study on human participants receiving an intervention to induce iso-osmotic diarrhea, a less extreme method to mimic a physiological disturbance of gut microbiome than antibiotic-induced cleanse, and revealed that rapid transit time affects microbiome composition. This study pointed out that although rapid transit, usually resulting from diarrhea, can cause dysbiosis, the gut microbiome communities revert to their pre-cleanout states within ~5 days after the cleanout, depending on the specific strains of bacteria (91). Because of the differences in transit time between people when sampling a single microbiome time point in a study, different durations of dietary history may be represented in a single stool sample for different people. In people who have a bowel movement 3 times per day one may observe diet-microbiome interactions at shorter time intervals, whereas individuals who have bowel movements less frequently, on the order of once a day or every other day, may have longer dietary time intervals represented in a single stool sample. More research is needed into how to best manage this temporal variation when analyzing diet-microbiome interactions. We have demonstrated that using several days of recent dietary history in models can improve the ability to pair diet and microbiome covariation (6). Other approaches to manage variation in transit time include the provision of foods labeled with dyes to measure transit time at the start of a study, or the ingestion of radio-opaque labels coupled with x-ray to empirically measure transit time. The latter has the distinct benefit of avoiding the ingestion of potentially microbiome-active food dyes, however it also comes with increased investigator and participant risk and burden.

**Figure 3:**
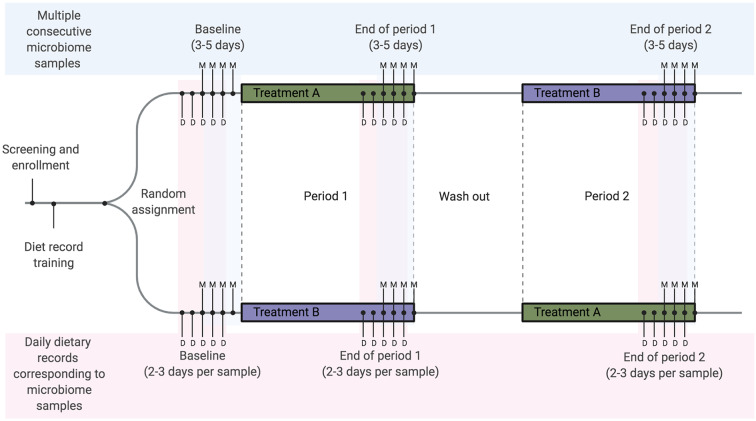
Example diet and microbiome sampling timeline for a longitudinal cross-over dietary intervention study. We recommend that diet-microbiome studies longitudinally follow individual participants and include multiple consecutive microbiome samples per study timepoint or period. As an example, a cross-over study design using repeated longitudinal sampling would include a total of 9–15 microbiome samples per person over three sampling time points. Here we show the mid-range of 4 samples per time point (labeled with M). Optimally, for each microbiome sample collected, 2–3 days of dietary records will be collected from each time point for a total of 15–18 dietary records over the study. In this example 2 days of records are collected prior to each sample (labeled with D). These records overlap, resulting in a total of 5 days of dietary records at each time point.

We recommend the collection of dietary recalls or records from 2-3 days prior to the sample collection period (Figure 3). In a cohort where transit time is measured and known it may be possible to optimize the dietary collection window relative to each stool sample to capture the dietary intake that best corresponds to each microbiome sample. When transit time is unknown or cannot be measured, then 3 days should be sufficient to capture most of the dietary intake from the days prior to a stool sample (6). Diet should be collected in a way that ultimately allows for the analysis of food choices, and not simply nutrient totals, to provide opportunity to assess data for food sources of nutrients and capture some of the dietary complexity and diversity that is not apparent from macronutrient totals. The timing of meals and other eating behaviors also impact gut microbiome composition (92). The microbiome responds to circadian rhythms, potentially driven by meal timing and fed-fast cycles (93). As this is not generally controllable in free-living human studies, fecal collection time should be recorded and used as a covariate in analysis to determine relationships relative to meal timing and fasting intervals.

## Recommendations When Controlling for Habitual Diet

Dietary intake is more variable than microbiome composition both within and between people (6), and studies of diet in the microbiome space must take this dietary variation into account whenever feasible. Measurement of dietary variation may be particularly important when studying diverse populations, because while dietary intake in the US is highly variable, dietary intake in other parts of the world may be homogenous from day-to-day and vary only over longer time periods (46, 47). Considerations for dietary measurement can vary depending on study design and outcomes measured. Considerations for different types of studies are included below.

Even if we are able to account for baseline microbiome differences with study design and pre-study microbiome-typing, we may also want to account for differences in habitual dietary intake at baseline. One way to control for variation in dietary intake in microbiome studies would be to switch participants onto an identical, standardized baseline diet prior to the intervention period. In practice, this is not trivial because the shift to the standardized diet can have its own effects on the microbiome, and thus may obscure the effects of the intervention. For example, Gurry et al. used a meal replacement beverage (Ensure) in an attempt to standardize the diet of their cohort prior to testing the effects of specific foods and supplements, but changing participants to this standardized beverage induced changes to their gut microbiomes including some diversity loss, and the changes were not consistent across all participants (94). From this study, it is difficult to determine whether the microbiome diversity loss was due to the colon cleanse which the study participants also underwent, or from the change to the standardized Ensure diet. This example raises several additional questions that are outside the scope of this article: Should a control diet be representative of habitual intake? What should a standardized baseline diet consist of in terms of macronutrient and micronutrient composition and food diversity? Are there ways to more accurately represent typical diets made of real food by considering more than just macronutrient and micronutrient composition? If so, what features should be considered? Should control diets be liquid meal replacements or standardized whole-food diets?

Based on currently available information, we recommend controlling variation in dietary intake by *stabilizing* diet, rather than by *standardizing* diet. This ensures consistency of individual diet during microbiome-focused intervention studies but does not attempt to modify habitual diet. The simplest approach is to ask participants to maintain their normal diet. More complex approaches would assign each subject to a specific constrained diet based on their own recent dietary intake data. Ultimately the best dietary approach will be study-dependent, and should be considered carefully when planning interventions to improve the ability to draw strong conclusions.

## Recommendations for Dietary Interventions

Dietary intervention studies are likely the best way to understand the impact of dietary components on the microbiome, but themselves come with some caveats. Cross-over intervention studies, where each participant undergoes both the target intervention and the control arm, with a washout period between treatments, is the most optimal study design for studying the effects of a dietary intervention because each participant acts as their own control. Cross-over designs have been successfully implemented in diet-microbiome studies (19, 24, 95), and allow the researcher to conduct a within-person comparison instead of a between-person comparison, reducing the sample size needed to detect differences by at least half, and reducing the confounding effects of inter-individual variation (96). Cross-over studies are more difficult to conduct than parallel studies for several reasons including longer study duration and higher participant burden, and it is imperative that the washout period be long enough to prevent carryover effects (97). Proper control of factors such as randomization of order, verifying a return to baseline diet during the washout period, and general compliance with study protocols over a longer study duration are critical and typically require a higher level of study personnel training and time. A lead-in period both prior to the start of the study and during the latter half of the washout period are also recommended to acclimate participants and to stabilize any relevant diet and lifestyle factors (98). Despite these obstacles, cross-over study designs hold promise for understanding the effects of diets on the gut microbiome due to their unique ability to not just account for or diminish the noise of inter-individual variability, but to allow the study of personal responses, which can lead the way to personalized diets, and personalized microbiome-oriented recommendations.

Many studies have assessed the intake of grains and grain products (99, 100), and whole foods including walnuts (101), almonds (102), broccoli (103) and other cruciferous vegetables (104), and pistachios (105) for their impact on the microbiome. The literature on whole-food interventions has been recently reviewed (106). These studies tend to reveal modest changes in microbiome structure. However, it is also interesting to consider how intervening with food products alters habitual intake and how changes to habitual diet can affect an intervention in ways that may not be fully appreciated.

The short-term impact of diet on the microbiome beta-diversity is well-known, while the finer details and specific impacts of individual foods are still poorly understood. Results from mouse models revealed that large changes in diet can cause rapid gut microbiome compositional changes within a small number of days (107, 108). In human studies, gut microbiome composition responds at a similar rate. For example, David et al. showed that dietary interventions change microbiome composition significantly starting only 1 day after the intervention reached the distal gut (23). Largely based on these data, dietary intervention studies use a minimum of 3 to 5 days for maintenance diet before intervention or between crossing-over diets (109). Other intervention designs, in contrast, apply longer maintenance diets between different interventions (19, 95, 99, 100, 110). The rationale used to determine dietary intervention length is not always justified, likely due to the lack of strong evidence for best practices in the field. It is still unclear how long dietary interventions would need to be to shift community membership such that even after removal of the intervention the microbes persist (111). In fact, some researchers have suggested that the inability of gut microbiome changes to persist could be an evolutionary advantage resulting from the constant switching of diets in the hunter-gatherer era (23, 112). Determining the length of dietary intervention when the outcome is not a microbiome change but rather a biomarker change in the host, will be more challenging. The length of an intervention required to see differences in host phenotype is likely on the order of weeks or months, depending on the markers of interest (111). This is particularly salient for cross-over intervention studies, since the duration of the washout period is critical to ensure that there are no carryover effects, which can confound the results.

An additional consideration for planning dietary intervention studies where study foods will be provided to the study participants is preference. It is imperative to know whether a study participant will be willing to eat all of the study foods and meals provided. Ideally, the study meal plans, with all the intended ingredients and preparation methods, should be clearly explained in advance, and potential participants should be asked during screening whether they are willing to follow all study protocols, to prevent the recruitment of non-compliant participants. If substitutions need to be made after the participant has already been recruited into the study, these substitutions may impact the effects of the intervention on the microbiome. For example, in a recent study we tested the effects of a Mediterranean style diet compared to a fast food diet of burgers and fries (24). Even in this small group of only 10 healthy, young subjects who reported to be omnivores and willing to consume foods provided to them, 3 of the participants did not like certain foods and ingredients and their preferences were accommodated to ensure that they were at least receiving the intended calorie levels and macronutrient breakdowns. One participant did not like chickpeas so his lunch salad contained black beans instead. Another participant did not like balsamic vinegar so his salad only contained olive oil instead of vinaigrette dressing. Yet another participant did not like walnuts so these were replaced with almonds in the mixed nut snack. While these substitutions seem reasonable in terms of maintaining consistent macronutrient and micronutrient levels, the poly- and oligosaccharide composition of the fermentable component of chickpeas and black beans, for example, may be different enough to elicit differential microbiome responses.

Dietary interventions with microbiome outcomes should include placebos and blinding where possible; these considerations are not unique to microbiome studies (113). When dietary interventions are implemented thought should be given to how adherence and compliance will be assessed. The best practice in feeding trials is to provide all meals to participants and to collect plate waste to determine true intake. In studies where that level of control is not possible, or when dietary interventions are implemented using instruction, researchers should include multiple dietary records throughout the study period to see how well-participants comply with recommended intake instruction. Furthermore, when designing feeding trials and interventions there are numerous axes of dietary intake beyond diet make-up in terms of foods and food quantity that ideally should be controlled. These include meal timing, length or duration, and location—all features that affect the food environment. Researchers should also consider capturing qualitative metrics surrounding hunger and satiety when they are relevant for specific dietary intervention questions.

Ultimately, the results of even extremely well-controlled dietary intervention studies are likely to be affected by personalized diet-microbiome interactions. The individual variation across people's microbiomes arises from a combination of factors beginning in infancy, likely including birth mode and antibiotic exposure (64, 65). Individual microbiome responses also likely stem from variation in biogeography along the gastrointestinal tract, stochastic or random events, and complex community interactions. For example, a recent well-controlled study measuring microbiome response to either fast food or the Mediterranean diet demonstrated individual variation. Most participants showed consistent and comparable changes in response to dietary intervention. However, there was a small number of participants whose microbiome shifts were in opposite directions when compared to the majority (24). Individual responses to interventions and dietary intake are documented across multiple studies (6, 16, 19). These kinds of observations create difficulties for analysis, particularly when using group means for comparisons, but there is strong evidence now that individual-specific responses should be expected and modeled.

## Improved Dietary Assessment Methodologies Could Improve Diet-Microbiome Understanding

Participant compliance and the cost of participant time remain an obstacle for researchers to attain authentic dietary information in a practical way. Improvement in dietary assessment by shortening the time of data collection and reducing participant workload would improve the overall quality of dietary intervention trials. Recent work has applied the technique of metabarcoding to evaluate the plant component of diet from stool samples using the *trn*L-P6 marker gene (114). Similar techniques have previously been used to assess diet in non-human primate populations (115, 116); demonstrating that DNA from plant species persists through digestion and can be identified in stool. While these methods may not be able to quantify protein or kilocalorie content, they should provide a method to identify plant diversity and could provide a non-invasive measure of diet quality. Importantly, plant diversity has previously been linked to microbiome features (73) and ways to quantify this may improve future studies of diet and the microbiome without increasing participant burden. Moreover, detection of dietary intake within stool samples will allow for perfect matching between dietary and microbiome features without concern for transit time or self-report.

Ideally, researchers could follow a meal through the gut and collect data about that meal and the microbial community changes that are induced by it. Technology that allows easier sampling throughout the gastrointestinal tract using capsules to capture information directly from within the small intestine and colon is a promising advance (117). The ideal approach, though costly and often impractical due to high participant burden, is to couple this type of measurement with the direct analysis of food composition and content from duplicate plates, which can provide precise estimates of exposure to specific components (118), and even dietary microbe information (60).

Most of the technology-based approaches for dietary assessment still depend on self-reported dietary intake (49). The computerization of the automated multiple pass method (AMPM) is commonly used for dietary recalls (119). In the US, this method is widely available to researchers using the Automated Self-Administered 24-h (ASA24) dietary assessment tool. ASA24 can be used to apply the AMPM method at scale in large studies as it has acceptable validity relative to interviewer administered recall (120). Numerous other web-based and computerized approaches for dietary assessment are available in other countries (49). Using a computer-administered approach such as ASA24 is not without limitations and ASA24 in particular may be most appropriate when studying computer-savvy adults (121, 122). Other exploratory technology such as image-based diet capture could improve dietary assessment and reduce participant burden. Feature recognition from images is difficult (123). Even informed humans struggle with food image recognition projects, with dietetics students accurately identifying foods from images 79.5% of the time (124). Image-assisted methods for dietary assessment with mobile and wearable technologies (125) to detect eating occasions, mastication, and hand-to-mouth gestures have great potential and will likely harness machine learning (126); we expect these approaches to continue to improve in accuracy in the coming decade. Efforts to capture and quantify dietary intake from photos taken with a smartphone or from a combination of cameras and wearable devices have not been applied extensively to microbiome datasets. Available dietary assessment technology options using these types of approaches and created for research purposes in the US include eButton (127), Remote Food Photography Method (128), and Technology Assisted Dietary Intake Assessment (125).

Given the still-emerging technologies in dietary assessment, exploration of alternative methods of collecting dietary data is an important area of future work. In the short-term, we may need to shift the paradigm in dietary assessment from a nutrition-centric perspective to a microbiologist-centric perspective and to design and validate a microbiome-focused food intake questionnaire. A tool like this should divide foods into groups that have particular relevance for the microbiome based on fiber type or phytocompound composition known or suspected to affect the microbiome. There may be other proxies that we can capture as latent variables for diet that discard previous dietary norms and assumptions such as macronutrients and kilocalories while still collecting key information relevant to microbiome analysis. There is likely also a role for nutritional biomarkers or blood metabolites to characterize individual diet-microbiome interactions, particularly when personalization is considered relative to microbiome changes (129). Combined, advances in these areas as well as in microbiome sequencing and analysis techniques have the potential to greatly improve the quality of diet-microbiome studies to move beyond correlational analysis and develop understanding of causative relationships.

## Future Directions and Conclusions

In this article, we have presented a variety of considerations including both practical recommendations that can be immediately incorporated into studies and aspirational recommendations that will require greater effort for implementation (Table 1). Ultimately, we believe that microbiome research will benefit from more rigor and more informed design in dietary assessment and intervention. We have also raised several questions that do not yet have good answers. Moving forward, the field is in need of more well-controlled longitudinal studies and controlled feeding trials that can isolate the impact of specific changes in dietary intake on microbial communities in human hosts. With well over 9,000 unique foods represented in most nutritional databases and an estimated 26,000 unique food chemicals in the larger food supply (38) it is difficult to determine which foods are the most promising candidates for intervention trials. Studies with strong dietary data collection methods will therefore play an important role in identifying potential diet-derived bioactive compounds and their food sources that can be investigated more closely with well-designed interventional studies.

**Table 1:** Summary of current and future recommendations for dietary factors in diet-microbiome studies.

	**Feasible now**	**Ideal now**	**Future needs**
Study design and sampling protocols	Include large sample sizes for cross-sectional studies (400–500 participants).	Dense longitudinal sampling during and after interventions.	Stratify longitudinal studies by baseline microbiome composition.
	Collect multiple fecal samples (e.g., for 3 consecutive days) per study time point. Design longitudinal studies and favor cross-over intervention studies over parallel study designs.	Collect more fecal samples (e.g., for up to 7 consecutive days) per study time point, or daily sampling throughout an entire study.	Sequence microbiome during recruitment to enroll predicted responders.
Dietary assessment	In addition to or in lieu of using food frequency questionnaires (FFQ), participants report food intake using multiple 24-h dietary recalls or 3-day diet records.	Participants report food intake using multiple 3-day dietary records paired with each microbiome sample collected and receive instruction or training preferably by a dietetic professional.	Measure biochemical markers for intake of specific foods or dietary components (e.g., plant DNA in stool, metabolomic markers in urine or blood) and use technology to accurately capture dietary intake.
Dietary intervention	Participants stabilize their diet by consuming a consistent diet (e.g., the same breakfast, lunch and dinner for 3 days) individualized and based on their habitual dietary intake, prior to and/or during each study time point or set of sample collections.	Intervention meals are consumed at the same time, location, and within the same length of time and compliance is assessed by weighing plate waste.	Participants consume all of their study foods at the research center, consume only foods provided by the study but take the food with them and receive specific instructions, or consume their own foods but generate duplicate plates from which food composition can be measured directly.
Dietary data analysis	Conduct analysis of nutrient intake and food intake in terms of food groups and healthy eating indices.	Include detailed longitudinal analysis of food intake using methods that account for the multivariate nature of dietary data and relationships between foods.	Connect dietary intake data to food databases that contain extensive information about foods and food components and use machine learning approaches to compare with microbiome data.

*Table 1 highlights suggestions for improving studies investigating relationships between diet and the gut microbiome. We include suggestions that can be implemented currently with minimal additional resources, those that would be ideally implemented but require additional resources, and those that could be implemented in the future after further research and development*.

## Author Contributions

AJ wrote the manuscript and created the figures. JZ, JK, and AS wrote sections of the manuscript and assisted with figure planning. DK and AZ planned the review and contributed to editing and revising the manuscript. All authors read and approved the submitted version. All figures were created with BioRender.com.

## Conflict of Interest

AJ and DK have received research support from General Mills. AJ has received speaking fees from Abbott Nutrition. DK serves as CEO of CoreBiome, a company involved in the commercialization of microbiome analysis. CoreBiome is now a wholly owned subsidiary of OraSure. These interests have been reviewed and managed by the University of Minnesota in accordance with its Conflict of Interest policies. The remaining authors declare that the research was conducted in the absence of any commercial or financial relationships that could be construed as a potential conflict of interest.
